# Some natural hypomethylating agents in food, water and environment are against distribution and risks of COVID-19 pandemic: Results of a big-data research

**DOI:** 10.22038/AJP.2022.19520

**Published:** 2022

**Authors:** Mohammad Reza Besharati, Mohammad Izadi, Alireza Talebpour

**Affiliations:** 1 *Department of Computer Engineering, Sharif University of Technology, Tehran, Iran*; 2 *Quran Miracle Research Institute, Shahid Beheshti University, Tehran, Iran*; 3 *Department of Computer Science and Engineering, Shahid Beheshti University, Tehran, Iran*

**Keywords:** COVID-19, Nutrition, Risk, Survey, Hypomethylating agents, Big data

## Abstract

**Objective:**

This study analyzes the effects of lifestyle, nutrition, and diets on the status and risks of apparent (symptomatic) COVID-19 infection in Iranian families.

**Materials and Methods:**

A relatively extensive questionnaire survey was conducted on more than 20,000 Iranian families (residing in more than 1000 different urban and rural areas in the Islamic Republic of Iran) to collect the big data of COVID-19 and develop a lifestyle dataset. The collected big data included the records of lifestyle effects (*e.g.* nutrition, water consumption resources, physical exercise, smoking, age, gender, health and disease factors, etc.) on the status of COVID-19 infection in families (*i.e.* residents of homes). Therefore, an online self-reported questionnaire was used in this retrospective observational study to analyze the effects of lifestyle factors on the COVID-19 risks. The data collection process spanned from May 10, 2020 to March 19, 2021 by selecting 132 samples from more than 40 different social network communities.

**Results:**

The research results revealed that food and water sources, which contain some natural hypomethylating agents, mitigated the risks of apparent (symptomatic) COVID-19 infection. Furthermore, the computations on billions of permutations of nutrition conditions and dietary regime items, based on the data collected from people’s diets and infection status, showed that there were many dietary conditions alleviating the risks of apparent (symptomatic) COVID-19 infection by 90%. However, some other diets tripled the infection risk.

**Conclusion:**

Some natural hypomethylating agents in food, water, and environmental resources are against the spread and risks of COVID-19.

## Introduction

Since World War II, the COVID-19 pandemic has been the most catastrophic event worldwide. Many researchers are trying to solve the multifaceted complex aspects of this pandemic. Lifestyle (and its effects on the risks of a pandemic) is among these aspects (Mutch, 2020 ▶). 

Researchers argue that there are some lifestyle and dietary factors that can affect the risks of SARS-CoV-2 infection such as physical activities, a current habit of smoking, a former habit of smoking, intake of vitamins (*e.g.* D, A, K, B and E), intake of micronutrients (*e.g. *zinc, magnesium, copper, and ferritin), natural bioactive antiviral chemicals (*e.g.* emodin and curcumin), and natural antimicrobial and anti-inflammation foods (*e.g.* natural honey, pepper, garlic, and ginger) (Calder, 2020 ▶; Siahpoosh, 2020 ▶). 

An online self-reported questionnaire was used in this retrospective observational study to analyze the effects of lifestyle factors on COVID-19 risks. The big data and numerical method help address the problem accurately (according to the precision medicine and other modern approaches). The relative risk of each factor was determined separately and comparatively. After that, the combination risks of dietary factors (*i.e.* different dietary conditions and regimes) were determined.

The research results show that lifestyle and nutrition factors had relatively significant impacts on the risks of the apparent (symptomatic) COVID-19 infection. In particular, some natural hypomethylating agents (El-Hussein et al., 2018) in food, water, and environmental resources are against the spread and risks of the COVID-19 pandemic.

## Materials and Methods

A relatively extensive questionnaire survey was conducted on more than 20,000 Iranian families (residing in more than 1000 different urban and rural areas in the IRI) to collect big data on COVID-19 and create a lifestyle dataset (with more than 1M data records and more than 1G items obtained from the semantic entailment rules). The collected big data included records regarding the lifestyle effects (*e.g.* nutrition, water consumption resources, physical activities, smoking habits, age, gender, health and disease factors, and many other items) on the status of the COVID-19 infection in families (*i.e.* residents of homes). 

**Sampling and participants**

An online self-reported questionnaire was designed in this retrospective observational study to inquire about the status of family members considering COVID-19. The infection status was diagnosed as “non-infection”, “suspected infection”, “definitive/apparent infection”, and “SARS-CoV-2 death”. To view the questionnaire and all of its items, you can refer to it (Besharati, 2020 ▶).

In three different phases during the first, second, and third COVID-19 peaks in Iran in spring, summer, autumn and winter of (2020–2021), self-reported data regarding lifestyle and COVID-19 were collected from more than 20,000 Iranian families living in more than 1000 urban and rural areas. The data collection process spanned from May 10, 2020 to March 19, 2021 by selecting 132 samples from more than 40 different social network communities (*i.e. *channels with aggregately more than one million subscribers).

The results include a large body of data pertaining to the lifestyle and COVID-19 with more than one million data records and more than two billion information records.

## Results

**Findings about natural hypomethylating agents**

According to the research results, some of them are provided in Tables 1, 2, and 3, food and water resources which contain some natural hypomethylating agents can mitigate the risk of the apparent COVID-19 infection. They can be introduced as below: 

1**- **Some phytochemicals such as curcumin (found richly in turmeric) and trigonelline (found richly in coffee, fenugreek seed, and in lower concentrations, in fruits, vegetables, and natural honey). In addition to their hypomethylating effects, these natural bioactive phytochemicals act as anti-oxidative and anti-inflammatory agents; therefore, they can resist against the oxidative stress and cytokine storm which play major roles in severe phases of COVID-19 infection (*e.g. *lung injuries, Internal bleeding, *etc.*). 

2- Some metals and minerals (*e.g.* calcium, magnesium, zinc, *etc.*) 

3-Some naturally-occurring bio-concentrations of heavy metal ions in food, water, and environmental resources. 

4- Islamic fasting for some consecutive days, which had hypomethylating and anti-inflammatory effects (Mindikoglu et al., 2020 ▶) and in our data, it was observed to mitigate the risk of the apparent COVID-19 infection. 

5- Natural honey and its bioactive material. 

6-* Trigonella foenum-graecum* and its products (fenugreek seeds).

**Figure 1:**
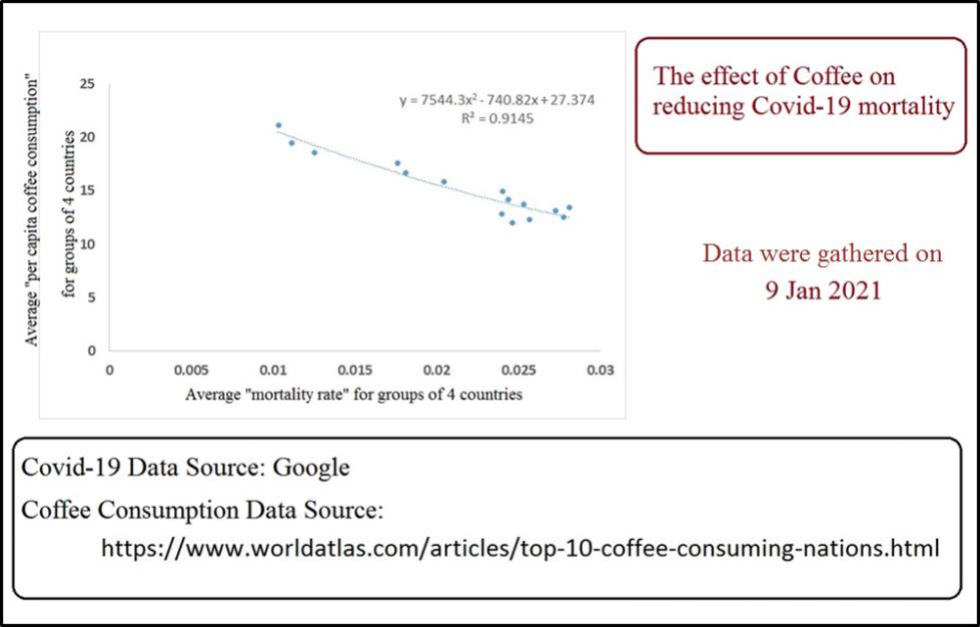
The effect of coffee on reducing COVID-19 mortality in groups of 4 countries. We considered only the countries with more than 5 kg per-capita annual consumption of coffee

7- Different types of coffee, especially the intense Arabic coffee, and any sources of trigonelline (Note: some countries with very high and intense consumption rates of coffee (*e.g.* Laos, Luxembourg, Qatar, and Oman) have had very low rates of fatality in the COVID-19 pandemic). There is a correlation between coffee consumption and reduced rate of fatality in the countries with coffee consumption rates of more than 5 kg per capita; Figure 1). 

8- The grape syrup and fruit roll-ups, fruit leather, and any sources of trigonelline and diverse alkaloids. 

9- Some types of tea and any sources of diverse alkaloids.

In addition to the bioactive materials, the research results indicate that the following foods, vitamins, and minerals mitigated the risk of the apparent COVID-19 infection: Probiotic dairy products; Vitamin D; Natural sources of vitamin C; Some natural sources of vitamin B3 (excluding fish); and Real sea salt (neither purified, nor filtered).

Finally, the research findings revealed that the following foods would greatly intensify the risk of the apparent COVID-19 infection: Sugar substitutes and artificial sweeteners; fish and some other sources of phosphorous and phosphate (e.g. residues of phosphate fertilizers in some fruits, foods, and surface runoff waters); sugar; soft drinks and soda (rich in phosphorous, anti-calcium, and sugar); fast food; pumpkin; deep-fried food; Western diet and other unhealthy diets. 

People who protected their bodies from mild influenza and common cold infections in the previous fall were highly prone to the risk of infection in the resultant data.

According to the computation results of billions of permutations of nutritional conditions and dietary regimes based on the data collected from the dietary and infection status of participants, there were many dietary conditions mitigating the risks of the apparent SARS-CoV-2 infection by 90%, whereas there were some dietary conditions increasing the risks by a factor of 3 or more.

In Table 1, the detailed results (for phase-1 of survey) are provided. Some items are added after initiation of the survey and during it, so the number of gathered questionnaires are different. According to our observations, the green rows are lowering the risk of COVID-19 apparent infection, and according to our observations, the red rows are increasing the risk of COVID-19 apparent infection.

**Table 1:** Results of phase 1 of data collection, for more than 11000 families

Factor in family life-style	Observed COVID-19 apparent infection risk change (in %)	Relative risk (RR)	Statistical significance due to 99.9% confidence interval	Statistical significance due to 95% confidence interval	Number of questionnaires
**Turmeric**	-87	0.45	yes	yes	more than 11k
**Black pepper**	-65	0.51	yes	yes	more than 11k
**Islamic fasting for entire ramadan**	-61	0.55	yes	yes	about 10k
**Cinnamon**	-59	0.55	yes	yes	more than 11k
**Legume and chickpea**	-52	0.55	yes	yes	about 5k
**Dark chocolate, dark cocoa**	-50	0.54	yes	yes	about 3k
**Bell pepper**	-48	0.59	yes	yes	more than 11k
**Tea**	-48	0.66	yes	yes	more than 11k
**Sea salt**	-48	0.57	yes	yes	about 10k
**Vitamin d or multivitamin tablets**	-46	0.62	yes	yes	more than 11k
**Walnuts or nuts**	-46	0.62	yes	yes	more than 11k
**Consuming rose water once a few days in food or drink**	-46	0.60	no	yes	about 5k
**Fruits natural products: grape syrup**	-45	0.61	yes	yes	about 5k
**Daily yogurt consumption**	-45	0.64	yes	yes	about 10k
**Tahini and natural products like it**	-44	0.61	yes	yes	about 10k
**Low or controlled consumption of oil**	-44	0.62	yes	yes	more than 11k
**Consume courgette once every ten days**	-44	0.59	yes	yes	about 5k
**Garlic**	-42	0.65	yes	yes	more than 11k
**Consume eggplant once every ten days**	-42	0.64	yes	yes	about 5k
**High consumption of fruits and vegetables**	-41	0.67	yes	yes	more than 11k
**Natural honey**	-41	0.67	yes	yes	more than 11k
**Green pea**	-38	0.63	no	no	about 5k
**Ginger**	-36	0.68	yes	yes	more than 11k
**Fruits natural products: fruit-roll**	-35	0.67	yes	yes	about 10k
**Local dairy products**	-33	0.71	yes	yes	more than 11k
**Daily coffee consumption**	-33	0.69	yes	yes	more than 11k
**Traditional breads (whole-wheat flour)**	-32	0.71	yes	yes	more than 11k
**Soybean and its products**	-32	0.70	no	yes	about 5k
**Head cabbage**	-31	0.69	no	yes	about 10k
**Islamic fasting, once a week**	-31	0.69	no	no	about 10k
**Vegetarian diet**	-29	0.71	no	no	about 10k
**Probiotic dairy products**	-25	0.76	no	yes	more than 11k
**Slimming weight loss diet or low-calorie diet**	-24	0.77	no	no	about 10k
**Non-alcoholic beer**	-21	0.79	no	no	about 5k
**Physical exercise and walking**	-19	0.82	no	yes	more than 11k
**Tobacco and smoking**	-15	0.86	no	no	more than 11k
**High consumption of apple juice or apple**	-14	0.86	no	no	about 5k
**Home water purification devices**	21	1.23	no	yes	more than 11k
**High consumption of deep frying or fried foods**	24	1.25	no	yes	more than 11k
**Soft drinks and soda**	24	1.25	no	no	about 3k
**High consumption of sugar**	26	1.27	no	no	about 3k
**Monthly consumption of fish meat or seafood**	27	1.31	yes	yes	more than 11k
**Weekly consumption of fish meat or seafood**	28	1.30	yes	yes	more than 11k
**High consumption of sweet pepper (not bell pepper, excluding bell pepper)**	37	1.37	no	yes	about 10k
**Eat fish meat or seafood once every two or three days**	42	1.44	yes	yes	more than 11k
**High consumption of fast food**	45	1.46	yes	yes	more than 11k
**Pumpkin**	49	1.5	no	yes	about 3k
**Sugar substitute, artificial sweeteners**	60	1.62	yes	yes	more than 11k

**Table 2:** Results of phase 1 and 2 of data collection, for more than 15000 families. Between may 2020 and august 2020, about 15,000 questionnaire forms of family lifestyle and COVID-19 state were collected. Please note that for some technical reasons, in this Table, RR is defined as: the ratio of the probability of an outcome in an exposed group to the probability of an outcome in the entire community

**Item in family life-style**	**Relative risk** **(RR) for apparent COVID-19 infection**	**Statistical significance due to 95% confidence interval**
Sugar substitute, artificial sweeteners	1.439662	yes
High consumption of fast food	1.412777	yes
Eat fish meat or seafood once every two or three days	1.262527	yes
High consumption of deep frying or fried foods	1.205496	yes
Weekly consumption of fish meat or seafood	1.200414	yes
Soft drinks and soda	1.166497	yes
Home water purification devices	1.164635	yes
Monthly consumption of fish meat or seafood	1.094933	yes
Municipal tap drinking water	0.930306	yes
Daily tea consumption	0.914711	yes
Natural sources of vitamin c	0.895762	yes
Iranian traditional bread (whole-wheat flour, namely sangak bread)	0.88815	yes
Tabriz cheese (a famous traditional\local dairy product)	0.882122	yes
Low consumption of fresh fruits and vegetables	0.862301	yes
Physical exercise and walking	0.85225	yes
Consumption of curry spices	0.846108	yes
Tobacco and smoking	0.842229	yes
Natural honey	0.840308	yes
Consume more bread than rice	0.838445	yes
High consumption of fresh fruits and vegetables	0.832401	yes
Daily yogurt consumption	0.824463	yes
Broth consumption (weekly)	0.81267	yes
Consumption of traditional bread other than sangak bread	0.805427	yes
Consumption of traditional herbal decoctions	0.801104	yes
Ginger	0.800078	yes
Consumption of local dairy products (usually)	0.796525	yes
Garlic	0.785149	yes
Vitamin d tablets, multivitamin tablets or other dietary supplements	0.779102	yes
Walnuts or nuts	0.777078	yes
Consumption of traditional bread	0.771945	yes
Islamic fasting for entire ramadan	0.771563	yes
Fruits natural products: fruit-roll	0.755408	yes
Consume eggplant once every ten days	0.75151	yes
Soybean and its products (weekly)	0.745369	yes
Head cabbage	0.743139	yes
Cinnamon	0.74085	yes
Consumption of probiotics dairy products (usually)	0.740696	yes
High consumption of legume and chickpea	0.734784	yes
Turmeric	0.732947	yes
Fruits natural products: grape syrup	0.730504	yes
Red pepper	0.71724	yes
Low or controlled consumption of oil	0.712104	yes
Tahini and natural products like it	0.706797	yes
Dark chocolate, dark cocoa	0.703308	yes
Bell pepper	0.700019	yes
Black pepper	0.689468	yes
Daily coffee consumption	0.679084	yes
Egg consumption (once or every two days)	0.677557	yes
Consume courgette once every ten days	0.665738	yes
Sea salt	0.657723	yes
Green bell pepper	0.629236	yes
Fasting in some days of sha'ban or rajab	0.618743	yes
Consuming rose water once a few days in food or drink	0.616927	yes

**Dietary regimes**

The collected dataset was employed to analyze the relative risks of different diets. In fact, a diet is a set of conditions for lifestyle and nutrition. In this study, each diet consisted of a set of four conditions, *i.e.* having an alpha item, having a beta item, lacking a gamma item, and lacking a zeta item. Instead of alpha, beta, gamma, and zeta items, nearly 100 other items can be placed in the lifestyle and COVID-19 questionnaire. Therefore, regarding the compositional permutation, this betting mode can be defined as one hundred million diets. If other items of the questionnaire (*e.g.* as ancestral ethnicity) are included in this permutation, the order of two billion diets can be defined, and the relative risk can be determined.

An important real example of the calculations and the results are presented in this section. The collected and processed big data included billions of operations showing that some diets reduced the infection risk by one-tenth. However, some other diets tripled the infection risk. Regarding these two numbers, only the diets given to at least more than 400 families in Tehran were included in their lifestyles; therefore, the diets were not abandoned. The calculation is based on the data collected from May 2020 to August 2020 in relation to more than 3000 families living in Tehran. For instance, according to the calculation based on the collected big data, “daily consumption of yogurt + daily consumption of coffee + no weekly consumption of fish + no excessive consumption of fast food” reduced the infection risk by one-tenth.

Our results suggest that turmeric and natural honey in dietary life style could reduce SARS-CoV-2 mortality ratios in families. Some details are accessible in a separate report (Besharati et al., 2021f ▶).

**Machine learning on relative risks of ethnicity diets**

Machine learning was employed to develop a model for the prediction of the “relative risk of different ethnic groups” from the “relative risk of diet items in different ethnic groups” with an accuracy of more than 85%. The calculation details are accessible in a separate report (Besharati et al., 2021b ▶). Accordingly, the relative risk of diet items can be related to the relative risk of their consuming groups. Hence, the infection risk of a group was a function of its diet (with an accuracy of 85%). Moreover, the other effective factors were excluded from calculations, although it does not mean that they left no effects.

**Table 3:** Results of phase 3 of data collection, for more than 5000 families. Please note that for some technical reasons, in this Table, RR is defined as: the ratio of the probability of an outcome in an exposed group to the probability of an outcome in the entire community

**Item in family life-style**	**Relative risk ** **(** **RR) for apparent COVID-19 infection**	**Statistical significance due to 95% confidence interval**
Cough in previous years	1.249814	yes
Gastrointestinal disorders	1.204284	yes
High consumption of fast food	1.17625	yes
Soft drinks and soda	1.160428	yes
Excessive consumption of sugar	1.140422	yes
High consumption of deep frying or fried foods	1.138614	yes
Cold friend	1.134633	yes
Excessive consumption of salt or saline substances	1.115845	yes
High consumption of fresh fruits and vegetables	0.948763	yes
Islamic fasting for entire ramadan (asked in a period of time that is not including ramadan)	0.941575	yes
Consumption of local dairy products (usually)	0.939141	yes
Natural honey	0.937049	yes
Ginger	0.926274	yes
Consumption of traditional herbal decoctions	0.924588	yes
Turnip	0.92219	yes
Natural sources of vitamin c	0.920392	yes
Consume eggplant once every ten days	0.919945	yes
Consume more bread than rice	0.919118	yes
Consumption of dates	0.916372	yes
Consumption of curry spices	0.91159	yes
Turmeric	0.904948	yes
Cinnamon	0.897708	yes
Broth consumption (weekly)	0.89722	yes
Garlic	0.895731	yes
Fruits natural products: fruit-roll	0.895142	yes
Consume courgette once every ten days	0.894557	yes
Tahini and natural products like it	0.890716	yes
Fasting in some days of sha'ban or rajab	0.889585	yes
Egg consumption (once or every two days)	0.886981	yes
Black pepper	0.883633	yes
Walnuts or nuts	0.878356	yes
Fruits natural products: grape syrup	0.87199	yes
High consumption of apples or apple juice	0.863573	yes
Tobacco and smoking	0.860466	yes
High consumption of legume and chickpea	0.850285	yes
Low or controlled consumption of oil	0.838262	yes
Consume olive or olive oil	0.837602	yes
Pumpkin	0.834565	yes
Bell pepper	0.831265	yes
Consumption of probiotics dairy products (usually)	0.830798	yes
Red pepper	0.819943	yes
Green tea	0.819119	yes
Sea salt	0.816141	yes
Consumption (at least weekly) of fenugreek or its products	0.814778	yes
Head cabbage	0.812343	yes
Green bell pepper	0.807073	yes
Consuming rose water once a few days in food or drink	0.788751	yes
Consumption of chicory sweat drink	0.783346	yes
High consumption of pomegranate in autumn	0.78079	yes
Fennel or its edible products (weekly consumption)	0.729659	yes

Note: Between september 2020 and march 2021, about 5,000 questionnaire forms of family lifestyle and COVID-19 state were collected. Questions about some new diet items (which were not in the previous version of questionnaire, such as the pomegranate) were added and the questionnaire form was updated.

**Risk-reducing and -increasing items based on the regression analysis**

To conduct these analyses, a previously described method (Besharati et al., 2021d ▶) was employed on the Turin National Super-Computing Platform (IPM, 2021). According to the regression analysis of features and topological data analysis, the items playing central roles in mitigating the risk of SARS-CoV-2 were introduced as bell peppers, turmeric, natural honey, dates, olive oil, garlic, “weekly broth consumption”, natural sources of vitamin C, “low and controlled consumption of oils”, walnuts and nuts, fruit roll-ups, stewed squash (non-fried), eggplant, fasting, coffee, and “fennel and its edible products”. 

Moreover, the items increasing the apparent COVID-19 infection risk were introduced as consumption of soft drinks, sugar, fried food, fast food, artificial and diet sweeteners, high consumption of fish, and even high consumption of chicken, according to the results of this observational study. 

**Consumption ratio of items between infection and no infection groups**

**Figure 2:**
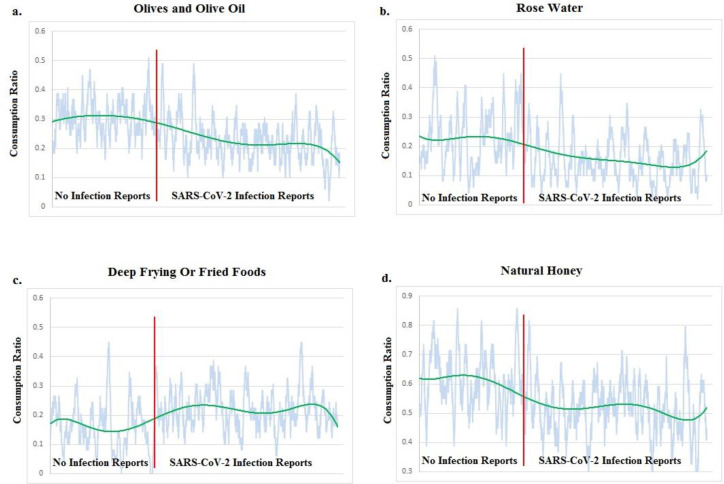
For a-b-d, the members of “No Infection Group” consumed the item more than the members of “Infection Group”. So the item may be protective. For c, the members of “No Infection Group” consumed the item less than the members of “Infection Group”. So the item may be risk-full

We compared the trends of running averages of consumption (the calculated consumption ratio for a window of 50 participants) for transition between infection and no infection groups (Figure 2). The depicted curve is like a frequency response function (FRF) _a function used to quantify the response of a system to an excitation_. Here, the excitation is an ordered transition from no infection reports to peak 1 infection reports, then peak 2, then peak 3, then peak 4 and then peak 5 infection reports. Again here, the response is the fluctuations of consumption ratio of nutrition items. So, an ascending response curve could be associated with a risk-full nutrition item. A descending response curve could be associated with a protective nutrition item. The gap value between the no infection group average-consumption-ratio (ACR) and infection group ACR could be considered the gain (or power) of response. Natural honey, olives and olive oil, head cabbage, rose water, tahini, garlic, dates sap, grape sap, bell pepper, curcumin, fenugreek and coffee are some examples of protective high-gain items. Soft drinks (soda), fried food and fast foods are examples of risk-full high-gain items.

**Thyroid and SARS-CoV-2**

Based on the evidence from the collected big data and other data from Iran and other countries, it appears that there is a significant relationship between the level of thyroid hormones and the risk of death due to the SARS-CoV-2 infection. This relationship is used in the prognosis of death. For more details, please refer to its separate report (Besharati et al., 2021c ▶). 

**Docking study**

**Figure 3:**
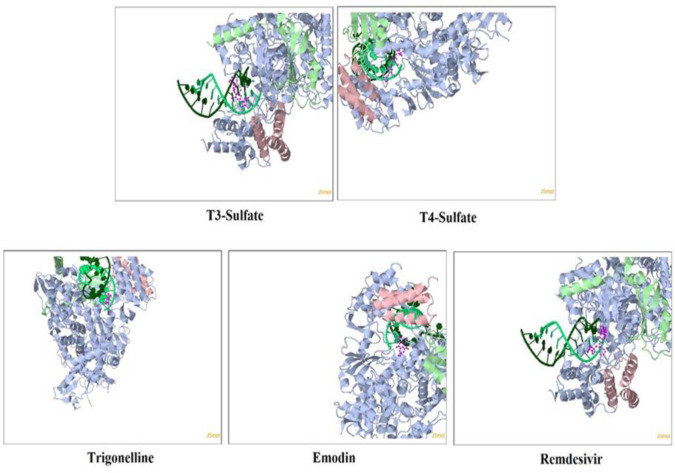
Docking Models for RdRp (RTP site)

A molecular docking study showed that T3 and T4 hormones had comparable docking scores in comparison with remdesivir, trigonelline, and emodin (the COVID-19 Docking Server was used) (Kong et al., 2020 ▶) for binding to some COVID-19 proteins (Table 4 and Figure 3). Furthermore, the compound nicotinate mononucleotide (*i.e.* a derivative of trigonelline) with the formula of C11H15NO9P+ succeeded in inhibiting the RNA-dependent RNA polymerase (RdRp (RTP site)) protein in the novel coronavirus with a score value of -9.3 (kcal/mol). It acted better than the energy amount for the remdesivir molecule (-9.2 (kcal/mol)) in inhibiting the same protein. Since inhibition of this protein plays a major role in the inhibitory function of remdesivir against the novel coronavirus (Mindikoglu et. Al., 2020 ▶), nicotinate mononucleotide compound can be considered an alternative to remdesivir in inhibiting the virus.

As the molecular weight of nicotinate mononucleotide (336 g/mol) is nearly half that of Remdesivir (603 g/mol), it is better in terms of both protein adhesion and absorption capacity. The main precursor of nicotinate mononucleotide, *i.e. *trigonelline alkaloid, is a naturally-occurring plant secondary metabolite, and nicotinate mononucleotide itself is present in mammalian biomolecular pathways. Therefore, it is likely to be more available, more cost-effective, and more non-toxic, and can act better than remdesivir. 

**Proposed model**

A model was hypothesized to explain the results. Some natural hypomethylating agents (in their simple forms or complexes such as alkaloid-metal complexes and trigonelline compounds) were able to attach to viroporins (and other viral proteins) as well as the virus RNA to mark them, disrupt their functionalities, destroy their sequences, and hack the cybernetics of the viral information encoded in them. After all, the cargo mechanisms and cargo proteins acting naturally on the hypomethylating agents in a cell and its membrane (such as heavy metal pumps, arsenic pumps, and intercellular RNA transport mechanisms) managed to export and deport the hostile virus RNA from the cell cytoplasm. There are some reports regarding triiodothyronine (T3) reduction in some SARS-CoV-2 patients (Besharati et al., 2021c ▶). According to a rigorous machine learning classification, the T3-level proved to be the key control variable for fatal outcomes in reported metabolomics data of the patients. The lower levels of T3 are associated with unbalanced states of the immune system, and the down-regulation of T3 is associated with up-regulation of “CD4/CD8 ratio” (Besharati et al., 2021c ▶). This phenomenon could cause severe autoimmune reactions and be responsible for fatal outcomes of SARS-CoV-2. 

**Table 4:** The docking study results

	Molecular weight	Main protease		RdRp (RTP site)		Nsp14 (N7-MTase)	
	(gr/mol)	Score value (kcal/mol)	RF score value (pKd)	Score value (kcal/mol)	RF score value (pKd)	Score value (kcal/mol)	RF score value (pKd)
**Trigonelline (N-methyl nicotinate)**	137.14	-4.4	4.24	-6.10	4.47	-5.7	4.37
**Emodin**	270.24	-7.20	5.76	-8.5	5.7	-8.8	6.18
**T3 Hormone-sulfate-**	731	-6.70	5.32	-8.0	6.16	-8.5	6.62
**T4 Hormone-sulfate-**	856.9	-7.00	5.57	-7.90	5.88	-7.9	6.26
**Remdesivir**	602.6	-7.70	6.59	-9.20	6.78	-9.9	7.28
**Nicotinic acid ** **D-ribonucleotide (Nicotinate mononucleotide)**	336.21	-7.00	5.01	-9.30	5.29	-7.70	4.96
**Ethyl 2-[2-(aminocarbonyl)-4-chlorophenoxy] nicotinate**	320.73	-6.40	6.17	-7.30	7.29	-7.60	6.29

Hence, the people with lower levels of T3 in their blood samples are at higher levels of SARS-CoV-2 mortality risk. There is also evidence about the downregulatory effects of T3 on the pro-inflammatory mechanisms of the SARS-CoV-2. In fact, the pro-inflammatory cytokines IL-1β and IL-6, which are downregulated by induction of the triggering receptor expressed on myeloid cells 2 (TREM2) pathway, were downregulated by T3 and sobetirome in microglia and macrophages stimulated with the pro-inflammatory SARS-CoV-2 spike protein (Ferrara et al., 2021 ▶). The molecular docking study shows that T3 and T4 had comparable docking scores as opposed to remdesivir, trigonelline, and emodin (the COVID-19 Docking Server was used) for binding to some COVID-19 proteins. A linear regression model (with correlation coefficient 0.98) correlated Urinary Iodine Concentration (UIC) and SARS-CoV-2 mortality rates of 91 countries until February, 24 2021. This finding is worthwhile for our hypothesis, because the iodine metabolism is related to the thyroid state and functions. Age, diabetes, obesity, ethnicity, gender, genetics, person’s mood (Besharati et al., 2021e ▶), epigenetics, and environmental factors (*e.g.* pollution and ionizing-radiation) can affect people’s T3 blood levels. This finding can exactly explain the reported effects of those risk factors (Besharati et al., 2021c ▶). 

## Discussion

**Related works**

Many *in silico*, *in vitro*, and *in vivo* studies as well as some clinical trials indicated that phytochemicals (*e.g.* curcumin (Lee et al., 2016 ▶), trigonelline (Özçelik et al., 2011 ▶), emodin (Ho et al., 2007 ▶), and vanillin could act against various phases of virus entry, infection, and the consequences (*e.g.* inflammation (Ashrafizadeh et al., 2020 ▶; Seif et al., 2020 ▶) and oxidative stress (Costa et al., 2020 ▶; Wang et al., 2018 ▶). Some previous studies showed that zinc, Cu, or even ppb concentrations of arsenic could act against SARS-CoV and SARS-CoV-2 infections. Recent studies reported the presence of some risk factors or inhibiting agents of the SARS-CoV infection in the environment (Zoran et al., 2020 ▶; Ma et al., 2020 ▶; Ward et al., 2020 ▶; Fattorini and Regoli, 2020 ▶; Li et al., 2020 ▶; Frontera et al., 2020 ▶; Yao et al., 2020 ▶), food (Bousquet et al., 2021 ▶; Abdulah and Hassan, 2020 ▶) and diet (Horne and Vohl, 2020 ▶), water, and some natural (Yang et al., 2020 ▶) or pseudo-natural compounds (Zhang et al., 2020 ▶). The existence of some phylogenetic and ethnicity risk factors of the COVID-19 (Williamson et al., 2020 ▶; Zeberg and Pääbo, 2020 ▶; Gibson et al., 2020 ▶) indicated the probable role of biological and epigenetic systems (Mutch, 2020 ▶) in fighting the COVID-19. Traditional medicines (*e.g.* Iranian Traditional Medicine (ITM) (Kaveh et al., 2015; Siahpoosh, 2020 ▶; Bahramsoltani and Rahimi, 2020 ▶) and Traditional Chinese Medicine (TCM) (Zaman et al., 2020 ▶; Chan et al, 2020 ▶)) had some common aspects with the data-driven observations of this study.

The human Angiotensin-converting enzyme 2 (ACE2) transmembrane protein (Hoffmann et al., 2020 ▶) is also another factor that the natural polypeptides of metal-alkaloids can use to ban the entry point of the novel coronavirus into the cell. Zinc, Cu, and arsenic are some heavy metals that can construct polypeptides (Banerjee, 2014 ▶) from trigonelline and some other small molecules. 

Recent studies support the proposed model. The metabolomics analyses of the hospitalized COVID-19 patients in the USA (Seattle) (Su et al., 2020 ▶), China (Wuhan) (Wu et al., 2020 ▶), and some other countries (Besharati et al., 2021a ▶), reported in separate studies, showed that trigonelline in the patient’s blood had an RR<1 for the severity and death caused by the COVID-19. This can be interpreted as a protective effect of trigonelline (Other evidence about this issue is available in a separate report (Besharati et al., 2021a ▶).

There is also evidence about the downregulatory effects of trigonelline on the pro-inflammatory mechanisms of the SARS-CoV-2 as the pro-inflammatory cytokines IL-1β and IL-6, and tumor necrosis factor (TNF)-α, were downregulated by trigonelline (Chowdhury et al., 2018 ▶; Omidi-Ardali et al.,2019 ▶; Costa et al., 2020 ▶).

**Summary**

Due to the results of present study, the natural resources of hypomethylating agents in water, food, and the environment can reduce the risk of the COVID-19 pandemic. The results of previous studies support our findings with numerous internal and external evidence (Özçelik et al., 2011 ▶; Calder, 2020 ▶; Siahpoosh, 2020 ▶; Su et al., 2020 ▶; Wu et al., 2020 ▶; Chowdhury et al., 2018 ▶; Omidi-Ardali et al.,2019 ▶; Costa et al., 2020 ▶; Karimi et al., 2021 ▶; Bousquet et al., 2021 ▶).

**Figure 4:**
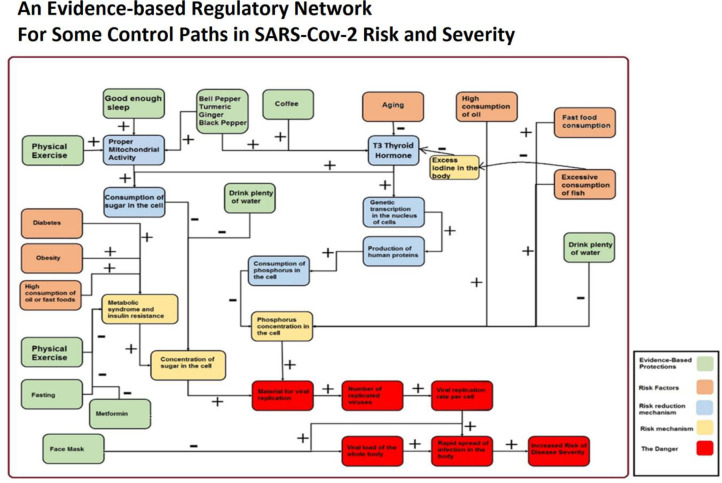
An overall summarization of our other findings in a regulatory network model

For an overall summarization of our other findings in a regulatory network model, see Figure 4. 

Considering the effects of trigonelline and compounds synthesized from it, the results of the risk analysis, ODDs ratios, and relative risks proposed that it can be 

used both as supplemental food and a candidate drug to conduct an intervention in the process of the COVID-19 prevention and treatment. 

The limitations in the process of designing and executing the present research included: 1: Limitation of the statistical population. Due to the limited financial resources available for the study, it was impossible to carry out a population census regarding all members of a statistical population. Therefore, voluntary random sampling was done on mobile social network platforms (based on self-motivated participation of the subjects). 2: Even though several time frames within almost a year (the different COVID-19 peaks) were used for data collection, the method could be improved by performing a comprehensive cohort study. 3: The researchers sought to use many data resources to conduct a surjective survey on different samples of the statistical population (i.e. Iran’s total population). However, one hundred percent surjection of the statistical survey cannot be guaranteed in terms of data diversity. Nonetheless, the internal and external evaluation of the data indicated their stratification and surjection of diversity. 4: Considering that the participants were volunteers and self-motivated, a very long questionnaire (with regard to the number of items) could not be used. The questionnaire included 100 items; however, a more comprehensive research can cover more items (i.e. several hundred items) on lifestyle and nutrition. 5: This research employed a method similar to that of other related studies and relied on the results obtained from the self-reports of participants. A small percentage of error is inevitable in this method. Our estimate based on internal and external evaluation of data accuracy suggested that the percent error and percent deviation caused by underreporting or misreporting were less than 5% of the total results.

The results of this research propose that nutrition and lifestyle are two important pivots or dimensions in fighting the COVID-19 pandemic. The natural hypomethylating agents in water and food together with lifestyle (exercising, fasting, and getting enough and proper sleep) can play a deciding role in the risk distribution of the spread of COVID-19. Specifically, trigonelline and the compounds synthesized from it can serve as a candidate in areas of research on prevention and treatment of COVID-19. The results of molecular docking (*in-silico* study) suggested that, the compounds synthesized from trigonelline could compete with the current first-line drugs of COVID-19 (especially, remdesivir in modern medicine and emodin in Chinese traditional medicine). It is worth noting that trigonelline with honey-water (i.e. a mixture of honey and water boiled over a gentle fire and then mixed with fenugreek herbal tea) was one of the prescriptions for treatment of pneumonia in Iranian-Islamic traditional medicine in past centuries acute respiratory syndromes epidemics.

Naturally, the results of this research alone cannot be used as a basis for general medical prescriptions by the regulating organizations during the COVID-19 pandemic, but, it can be considered a turning point in the course of SARS-Cov-2 and Phytomedicine studies. Taking into account the consistent results of previous studies and the present research, it can be expected that the regulating organizations dealing with the COVID-19 pandemic can conclude that use of natural hypomethylating agents in nutrition can be considered a centerpiece in the fight against the COVID-19 pandemic. Especially, the recommendation to consume materials containing trigonelline such as coffee, fenugreek seed herbal tea, bell pepper, anti-inflammatory spices, natural honey, fresh fruits-and-vegetables, rose water and some other available natural materials that, according to the results of studies, are effective in the distribution of the risks posed by COVID-19 in order to reduce the risks of apparent (symptomatic) infection.

There is some evidence that suggest the role of intake of iodine and thyroid hormones in the epidemiological status of SARS-CoV-2 (Besharati et al., 2021c ▶). There is a hypothesis that the cause of fatal outcomes of SARS-CoV-2 is related to thyroid hormones (Besharati et al., 2021c ▶).

The method employed in this research, which was based on big data and crowd-sourced contribution of people through social networks, can be used in other health studies (especially on communicable diseases and non-communicable diseases such as cancer, diabetes, metabolic syndrome, and even genetic diseases) to collect observational data. The main characteristics of this research method were its low cost, cost-effectiveness and stratified big-data. 

## Conflicts of interest

The authors have declared that there is no conflict of interest.

## References

[B1] Abdulah DM, Hassan AB (Relation of dietary factors with infection and mortality rates of COVID-19 across the world). 2020. J Nutr Health Aging.

[B2] Ashrafizadeh M, Rafiei H, Mohammadinejad R, Afshar EG, Farkhondeh T, Samarghandian S (Potential therapeutic effects of curcumin mediated by JAK/STAT signaling pathway: A review). 2020. Phytother Res.

[B3] Bahramsoltani R, Rahimi R (An evaluation of traditional Persian medicine for the management of SARS-CoV-2). 2020. Front Pharmacol.

[B4] Banerjee R (Inhibitory effect of curcumin-Cu (II) and curcumin-Zn (II) complexes on amyloid-beta peptide fibrillation). 2014. Bioinorg Chem Appl.

[B5] Besharati MR (2020). DAST Questionnaire. DiSysLab Sharif Technical Material.

[B6] Besharati MR, Izadi M, Talebpour A (2021a). Blood plasma trigonelline concentration and the early prognosis of death in SARS-Cov-2 patients (Version 10). Zenodo.

[B7] Besharati MR, Jafari N, Izadi M, Talebpour A, Hourali M (2021b). Relating SARS-Cov-2 infection risks to relative risks of dietary items in different ethnicities by machine learning (Version 8). Zenodo.

[B8] Besharati MR, Jafari N, Mostafavi E, Izadi M, Talebpour A (2021c). A hypothesis about the cause of fatal outcomes in SARS-Cov-2 (Version 11). Zenodo.

[B9] Besharati MR, Izadi M (2021d). SimulaD: A novel feature selection heuristics for discrete data.

[B10] Besharati MR, Izadi M, Talebpour A (2021e). People's mood and SARS-Cov-2 mortality (Version 2). Zenodo.

[B11] Besharati MR, Izadi M, Talebpour A (2021f). Turmeric and natural honey in dietary life style and SARS-CoV-2 infection and mortality ratios (Version 1). Zenodo.

[B12] Bousquet J, Anto JM, Czarlewski W, Haahtela T, Fonseca SC, Iaccarino G, Blain H, Vidal A, Sheikh A, Akdis CA, Zuberbier T (Cabbage and fermented vegetables: From death rate heterogeneity in countries to candidates for mitigation strategies of severe COVID‐19). 2021. Allergy.

[B13] Calder PC (Nutrition, immunity and COVID-19). 2020. BMJ Nutr Prev Health.

[B14] Chan KW, Wong VT, Tang SCW (COVID-19: An update on the epidemiological, clinical, preventive and therapeutic evidence and guidelines of integrative Chinese–Western medicine for the management of 2019 novel coronavirus disease). 2020. Am J Chin Med.

[B15] Chowdhury AA, Gawali NB, Munshi R, Juvekar AR (Trigonelline insulates against oxidative stress, proinflammatory cytokines and restores BDNF levels in lipopolysaccharide induced cognitive impairment in adult mice). 2018. Metab Brain Dis.

[B16] Costa MC, Lima TFO, Arcaro CA, Inacio MD, Batista-Duharte A, Carlos IZ, Spolidorio LC, Assis RP, Brunetti IL, Baviera AM (Trigonelline and curcumin alone, but not in combination, counteract oxidative stress and inflammation and increase glycation product detoxification in the liver and kidney of mice with high-fat diet-induced obesity). 2020. J Nutr Biochem.

[B17] El-Hussein MT, Power-Kean K, Zettel S, Huether SE, McCance KL (2018). Understanding Pathophysiology.

[B18] Fattorini D, Regoli F (Role of the chronic air pollution levels in the Covid-19 outbreak risk in Italy). 2020. Environ Pollut.

[B19] Ferrara SJ, Chaudhary P, DeBell MJ, Marracci G, Miller H, Calkins E, Pocius E, Napier BA, Emery B, Bourdette D, Scanlan TS (2021). TREM2 is thyroid hormone regulated making the TREM2 pathway druggable with ligands for thyroid hormone receptor. bioRxiv.

[B20] Frontera A, Cianfanelli L, Vlachos K, Landoni G, Cremona G (Severe air pollution links to higher mortality in COVID-19 patients: The “double-hit” hypothesis). 2020. J Infect.

[B21] Gibson WT, Evans DM, An J, Jones SJ (2020). ACE 2 coding variants: a potential X-linked risk factor for COVID-19 disease. bioRxiv.

[B22] Ho TY, Wu SL, Chen JC, Li CC, Hsiang CY (Emodin blocks the SARS coronavirus spike protein and angiotensin-converting enzyme 2 interaction). 2007. Antiviral Res.

[B23] Hoffmann M, Kleine-Weber H, Schroeder S, Krüger N, Herrler T, Erichsen S, Schiergens TS, Herrler G, Wu NH, Nitsche A, Müller MA (SARS-CoV-2 cell entry depends on ACE2 and TMPRSS2 and is blocked by a clinically proven protease inhibitor). 2020. cell.

[B24] Horne JR, Vohl MC (Biological plausibility for interactions between dietary fat, resveratrol, ACE2, and SARS-CoV illness severity). 2020. Am J Physiol Endocrinol Metab.

[B25] IPM (2021). Turin Cloud Services. Institute for Research in Fundamental Sciences.

[B26] Karimi M, Zarei A, Soleymani S, Jamali moghadam siahkali S, Asadi A, Shati M, Jafari M, Rezadoost H, Kordafshar G, Naghizadeh A, Mardi R (Efficacy of Persian medicine herbal formulations (capsules and decoction) compared to standard care in patients with COVID‐19, a multicenter open‐labeled, randomized, controlled clinical trial). 2021. Phytother Res.

[B27] Shahpar K, Chaichi Raghimi M, Sadr S, Abbassian A, Kaveh N, Choopani R (The role of Honeywater (Maul Asl) in the treatment of respiratory diseases from Iranian Traditional Medicine (ITM) point of view). 2015. Medical History.

[B28] Kong R, Yang G, Xue R, Liu M, Wang F, Hu J, Guo X, Chang S (COVID-19 docking server: A meta server for docking small molecules, peptides and antibodies against potential targets of COVID-19). 2020. Bioinform.

[B29] Lee HY, Kim SW, Lee GH, Choi MK, Jung HW, Kim YJ, Kwon HJ, Chae HJ (Turmeric extract and its active compound, curcumin, protect against chronic CCl 4-induced liver damage by enhancing antioxidation). 2016. BMC Complement Altern Med.

[B30] Li H, Xu XL, Dai DW, Huang ZY, Ma Z, Guan YJ (Air pollution and temperature are associated with increased COVID-19 incidence: a time series study). 2020. Int J Infect Dis.

[B31] Ma Y, Zhao Y, Liu J, He X, Wang B, Fu S, Yan J, Niu J, Zhou J, Luo B (Effects of temperature variation and humidity on the death of COVID-19 in Wuhan, China). 2020. Sci Total Environ.

[B32] Mindikoglu AL, Abdulsada MM, Jain A, Choi JM, Jalal PK, Devaraj S, Mezzari MP, Petrosino JF, Opekun AR, Jung SY (Intermittent fasting from dawn to sunset for 30 consecutive days is associated with anticancer proteomic signature and upregulates key regulatory proteins of glucose and lipid metabolism, circadian clock, DNA repair, cytoskeleton remodeling, immune system and cognitive function in healthy subjects). 2020. J Proteomics.

[B33] Mutch DM (The Covid-19 Global Pandemic: A natural experiment in the making). 2020. Lifestyle Genom.

[B34] Omidi-Ardali H, Lorigooini Z, Soltani A, Balali-Dehkordi S, Amini-Khoei H (Inflammatory responses bridge comorbid cardiac disorder in experimental model of IBD induced by DSS: protective effect of the trigonelline). 2019. Inflammopharmacology.

[B35] Özçelik B, Kartal M, Orhan I (Cytotoxicity, antiviral and antimicrobial activities of alkaloids, flavonoids, and phenolic acids). 2011. Pharm Biol.

[B36] Seif F, Aazami H, Khoshmirsafa M, Kamali M, Mohsenzadegan M, Pornour M, Mansouri, D (JAK inhibition as a new treatment strategy for patients with COVID-19). 2020. Int Arch Allergy Immunol.

[B37] Siahpoosh MB (How can Persian medicine (Traditional Iranian Medicine) be effective to control COVID-19?). 2020. Trad Integr Med.

[B38] Su Y, Chen D, Lausted C, Yuan D, Choi J, Dai C, Voillet V, Scherler K, Troisch P, Duvvuri VR, Baloni P (Multiomic immunophenotyping of COVID-19 patients reveals early infection trajectories). 2020. bioRxiv.

[B39] Wang ZY, Li Y, Chang WQ, Zheng JY, Li P, Liu LF, Xin GZ (Development and validation of a LC/MS-based method for the measurement of intracellular superoxide anion). 2018. Anal Chim Acta.

[B40] Ward MP, Xiao S, Zhang Z (Humidity is a consistent climatic factor contributing to SARS‐CoV‐2 transmission). 2020. Transbound Emerg Dis.

[B41] Williamson EJ, Walker AJ, Bhaskaran K, Bacon S, Bates C, Morton CE, Curtis HJ, Mehrkar A, Evans D, Inglesby P, Cockburn J (Factors associated with COVID-19-related death using OpenSAFELY). 2020. Nature.

[B42] Wu D, Shu T, Yang X, Song JX, Zhang M, Yao C, Liu W, Huang M, Yu Y, Yang Q, Zhu T (Plasma metabolomic and lipidomic alterations associated with COVID-19). 2020. Natl Sci Rev.

[B43] Yang W, Hu FL, Xu XF (Bee venom and SARS-CoV-2). 2020. Toxicon.

[B44] Yao Y, Pan J, Wang W, Liu Z, Kan H, Qiu Y, Meng X, Wang W (Association of particulate matter pollution and case fatality rate of COVID-19 in 49 Chinese cities). 2020. Sci Total Environ.

[B45] Zaman W, Saqib S, Ullah F, Ayaz A, Ye J (COVID‐19: Phylogenetic approaches may help in finding resources for natural cure). 2020. Phytother Res.

[B46] Zeberg H, Pääbo S (The major genetic risk factor for severe COVID-19 is inherited from Neanderthals). 2020. Nature.

[B47] Zhang G, Pomplun S, Loftis AR, Tan X, Loas A, Pentelute BL (Investigation of ACE2 N-terminal fragments binding to SARS-CoV-2 Spike RBD). 2020. bioRxiv.

[B48] Zoran MA, Savastru RS, Savastru DM, Tautan MN (Assessing the relationship between ground levels of ozone (O3) and nitrogen dioxide (NO2) with coronavirus (COVID-19) in Milan, Italy). 2020. Sci Total Environ.

